# 2-(4-Bromophenyl)-2-methyl­propan­amide

**DOI:** 10.1107/S1600536810009657

**Published:** 2010-03-27

**Authors:** Jian Wang, Hui Li, Hong Sun

**Affiliations:** a Department of Applied Chemistry, Yuncheng University, Yuncheng, Shanxi 044000, People’s Republic of China

## Abstract

In the crystal of the title compound, C_10_H_12_BrNO, inversion dimers linked by pairs of N—H⋯O hydrogen bonds generate *R*_2_^2^(8) loops. Further N—H⋯O hydrogen bonds link the dimers into sheets propagating in (100).

## Related literature

For the sythesis, see: Koltunov *et al.* (2004 ▶).
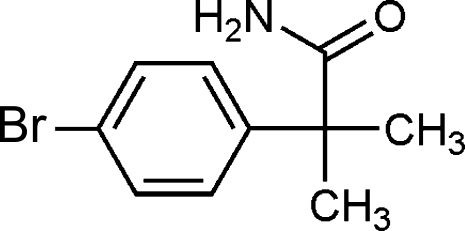


## Experimental

### 

#### Crystal data

C_10_H_12_BrNO
*M* *_r_* = 242.12
Monoclinic, 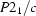

*a* = 16.425 (8) Å
*b* = 6.135 (3) Å
*c* = 10.152 (5) Å
β = 97.613 (7)°
*V* = 1013.9 (8) Å^3^
*Z* = 4
Mo *K*α radiation
μ = 4.01 mm^−1^
*T* = 113 K
0.20 × 0.18 × 0.12 mm

#### Data collection

Rigaku Saturn CCD diffractometer
Absorption correction: multi-scan (*CrystalClear*; Rigaku/MSC, 2005 ▶) *T*_min_ = 0.501, *T*_max_ = 0.644
9829 measured reflections
1787 independent reflections
1333 reflections with *I* > 2σ(*I*)
*R*_int_ = 0.090

#### Refinement

*R*[*F* ^2^ > 2σ(*F* ^2^)] = 0.035
*wR*(*F* ^2^) = 0.074
*S* = 0.99
1787 reflections
128 parameters
3 restraints
H atoms treated by a mixture of independent and constrained refinement
Δρ_max_ = 0.55 e Å^−3^
Δρ_min_ = −0.53 e Å^−3^

### 

Data collection: *CrystalClear* (Rigaku/MSC, 2005 ▶); cell refinement: *CrystalClear*; data reduction: *CrystalClear*; program(s) used to solve structure: *SHELXS97* (Sheldrick, 2008 ▶); program(s) used to refine structure: *SHELXL97* (Sheldrick, 2008 ▶); molecular graphics: *SHELXTL* (Sheldrick, 2008 ▶); software used to prepare material for publication: *CrystalStructure* (Rigaku/MSC, 2005 ▶).

## Supplementary Material

Crystal structure: contains datablocks I, global. DOI: 10.1107/S1600536810009657/hb5360sup1.cif

Structure factors: contains datablocks I. DOI: 10.1107/S1600536810009657/hb5360Isup2.hkl

Additional supplementary materials:  crystallographic information; 3D view; checkCIF report

## Figures and Tables

**Table 1:** Hydrogen-bond geometry (Å, °)

*D*—H⋯*A*	*D*—H	H⋯*A*	*D*⋯*A*	*D*—H⋯*A*
N1—H1*A*⋯O1^i^	0.89 (1)	2.12 (1)	2.990 (3)	167 (3)
N1—H1*B*⋯O1^ii^	0.88 (1)	2.12 (1)	3.002 (3)	173 (3)

Symmetry codes: (i) 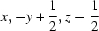; (ii) 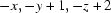.

**Fig. 1.**
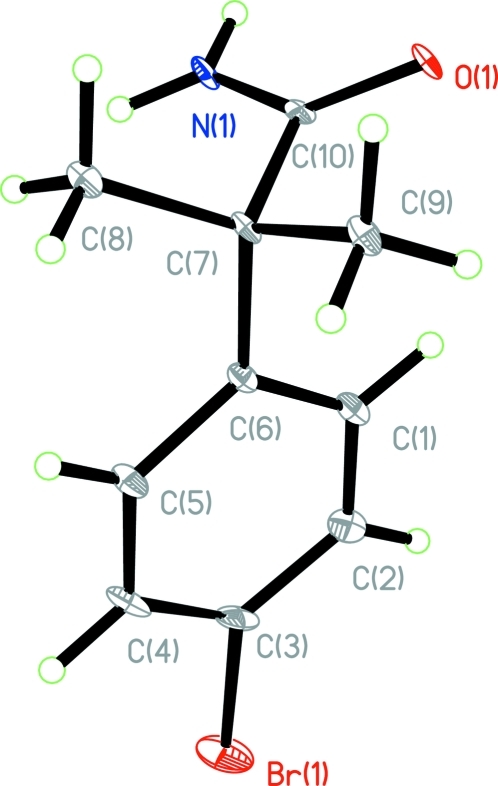
The molecular structure of (I). Displacement ellipsoids are drawn at the 30% probability level and H atoms are shown as spheres of arbitrary radius.

**Fig. 2.**
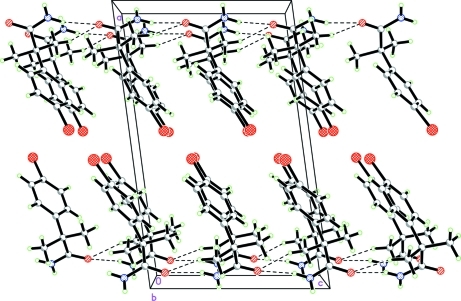
The crystal packing for (I).

## supplementary crystallographic information

### Comment

The reaction of amides towards weak nucleophiles such as nonactivated arenes have very broad utility in organic chemistry. However, little work has been done to investigate it. The title compound was synthesized by a facile method through the reaction of methacrylamide and benzene, catalyzed by AlCl_3_. In the crystal, the molecules are linked by intermolecular N—H···O hydrogen bonding interactions. Single-crystal X-ray diffraction analysis reveals that the title compound crystallizes in the Monoclinic space group *P* 21/c.

### Experimental

A mixture of AlCl_3_ (0.95 g, 7.1 mmol) and methacrylamide (0.2 g, 2.3 mmol) in benzene (3 ml) was stirred at 25 °C for 3 h, and was then poured over several grams of ice and extracted with CH_2_Cl_2_. The organic phase was separated, dried with anhydrous Na_2_SO_4_ and concentrated in vacuo to give a solid mixture of 2-methyl-3-phenylpropionamide and 2-methyl-2-phenylpropionamide (0.41 g, 97%) in 2:1 ratio. The title compound was separated by flash column chromatography on silica gel.  Colourless prisms of (I) were obtained by recrystallisation from ethanol.

### Refinement

The H atoms were positioned geometrically (C—H=0.95Å or 0.98 Å, N—H=0.88 Å) and refined as riding with U_iso_(H) = 1.2U_eq_(C,*N*) or 1.5U_eq_(methyl C).

### Crystal data

**Table:**

C_10_H_12_BrNO	*F*(000) = 488
*M**_r_* = 242.12	*D*_x_ = 1.586 Mg m^−^^3^
Monoclinic, *P*2_1_/*c*	Mo *K*α radiation, λ = 0.71073 Å
Hall symbol: -P 2ybc	Cell parameters from 3466 reflections
*a* = 16.425 (8) Å	θ = 2.2–27.9°
*b* = 6.135 (3) Å	µ = 4.01 mm^−^^1^
*c* = 10.152 (5) Å	*T* = 113 K
β = 97.613 (7)°	Prism, colourless
*V* = 1013.9 (8) Å^3^	0.20 × 0.18 × 0.12 mm
*Z* = 4	

### Data collection

**Table:**

Rigaku Saturn CCD diffractometer	1787 independent reflections
Radiation source: rotating anode	1333 reflections with *I* > 2σ(*I*)
multilayer	*R*_int_ = 0.090
Detector resolution: 14.63 pixels mm^-1^	θ_max_ = 25.0°, θ_min_ = 2.5°
ω and φ scans	*h* = −19→19
Absorption correction: multi-scan (*CrystalClear*; Rigaku/MSC, 2005)	*k* = −7→7
*T*_min_ = 0.501, *T*_max_ = 0.644	*l* = −12→12
9829 measured reflections	

### Refinement

**Table:**

Refinement on *F*^2^	Primary atom site location: structure-invariant direct methods
Least-squares matrix: full	Secondary atom site location: difference Fourier map
*R*[*F*^2^ > 2σ(*F*^2^)] = 0.035	Hydrogen site location: inferred from neighbouring sites
*wR*(*F*^2^) = 0.074	H atoms treated by a mixture of independent and constrained refinement
*S* = 0.99	*w* = 1/[σ^2^(*F*_o_^2^) + (0.0242*P*)^2^] where *P* = (*F*_o_^2^ + 2*F*_c_^2^)/3
1787 reflections	(Δ/σ)_max_ = 0.001
128 parameters	Δρ_max_ = 0.55 e Å^−^^3^
3 restraints	Δρ_min_ = −0.53 e Å^−^^3^

### Special details

**Table:**

Geometry. All esds (except the esd in the dihedral angle between two l.s. planes) are estimated using the full covariance matrix. The cell esds are taken into account individually in the estimation of esds in distances, angles and torsion angles; correlations between esds in cell parameters are only used when they are defined by crystal symmetry. An approximate (isotropic) treatment of cell esds is used for estimating esds involving l.s. planes.
Refinement. Refinement of *F*^2^ against ALL reflections. The weighted *R*-factor wR and goodness of fit *S* are based on *F*^2^, conventional *R*-factors *R* are based on *F*, with *F* set to zero for negative *F*^2^. The threshold expression of *F*^2^ > σ(*F*^2^) is used only for calculating *R*-factors(gt) etc. and is not relevant to the choice of reflections for refinement. *R*-factors based on *F*^2^ are statistically about twice as large as those based on *F*, and *R*- factors based on ALL data will be even larger.

### Fractional atomic coordinates and isotropic or equivalent isotropic displacement parameters (Å^2^)

**Table:**

	*x*	*y*	*z*	*U*_iso_*/*U*_eq_	
Br1	0.44964 (2)	0.60527 (5)	0.82615 (4)	0.03800 (16)	
O1	0.07026 (12)	0.3192 (3)	1.10663 (18)	0.0161 (5)	
N1	0.05185 (16)	0.3190 (4)	0.8836 (2)	0.0152 (6)	
C1	0.25495 (19)	0.4020 (4)	1.0149 (3)	0.0172 (7)	
H1	0.2230	0.4624	1.0773	0.021*	
C2	0.3202 (2)	0.5183 (5)	0.9799 (3)	0.0211 (7)	
H2	0.3331	0.6576	1.0180	0.025*	
C3	0.36720 (19)	0.4317 (5)	0.8887 (3)	0.0207 (7)	
C4	0.35071 (19)	0.2238 (5)	0.8383 (3)	0.0241 (8)	
H4	0.3844	0.1611	0.7792	0.029*	
C5	0.28481 (19)	0.1088 (5)	0.8747 (3)	0.0178 (7)	
H5	0.2737	−0.0335	0.8400	0.021*	
C6	0.23442 (18)	0.1968 (4)	0.9608 (3)	0.0124 (6)	
C7	0.15824 (18)	0.0770 (4)	0.9971 (3)	0.0122 (7)	
C8	0.12810 (19)	−0.1034 (4)	0.8969 (3)	0.0170 (7)	
H8A	0.1223	−0.0439	0.8066	0.026*	
H8B	0.0748	−0.1582	0.9159	0.026*	
H8C	0.1680	−0.2230	0.9044	0.026*	
C9	0.17838 (19)	−0.0249 (4)	1.1358 (3)	0.0168 (7)	
H9A	0.2222	−0.1331	1.1346	0.025*	
H9B	0.1292	−0.0960	1.1608	0.025*	
H9C	0.1966	0.0893	1.2005	0.025*	
C10	0.08953 (17)	0.2487 (4)	1.0001 (3)	0.0117 (6)	
H1A	0.0657 (16)	0.283 (4)	0.8050 (15)	0.025 (9)*	
H1B	0.0127 (13)	0.417 (4)	0.885 (2)	0.025 (9)*	

### Atomic displacement parameters (Å^2^)

**Table:**

	*U*^11^	*U*^22^	*U*^33^	*U*^12^	*U*^13^	*U*^23^
Br1	0.0253 (3)	0.0389 (3)	0.0549 (3)	−0.00663 (17)	0.0241 (2)	0.00685 (18)
O1	0.0196 (13)	0.0189 (11)	0.0122 (11)	0.0030 (9)	0.0113 (9)	−0.0008 (9)
N1	0.0169 (15)	0.0182 (14)	0.0121 (14)	0.0065 (12)	0.0079 (12)	−0.0018 (11)
C1	0.0176 (19)	0.0195 (17)	0.0161 (16)	0.0028 (14)	0.0078 (14)	0.0009 (13)
C2	0.0189 (19)	0.0212 (17)	0.0237 (18)	0.0021 (14)	0.0049 (15)	0.0023 (14)
C3	0.0107 (18)	0.0303 (19)	0.0229 (18)	−0.0003 (14)	0.0089 (14)	0.0090 (14)
C4	0.0163 (19)	0.0298 (19)	0.0294 (19)	0.0059 (15)	0.0151 (15)	0.0007 (15)
C5	0.0183 (19)	0.0183 (17)	0.0190 (17)	0.0011 (13)	0.0103 (14)	0.0003 (13)
C6	0.0131 (17)	0.0135 (15)	0.0115 (15)	0.0032 (13)	0.0047 (13)	0.0026 (12)
C7	0.0147 (18)	0.0117 (15)	0.0119 (15)	0.0027 (12)	0.0079 (13)	−0.0001 (12)
C8	0.0187 (19)	0.0152 (16)	0.0189 (17)	0.0012 (13)	0.0084 (14)	−0.0001 (13)
C9	0.021 (2)	0.0164 (16)	0.0146 (16)	0.0014 (14)	0.0087 (14)	0.0013 (13)
C10	0.0117 (16)	0.0101 (15)	0.0154 (16)	−0.0049 (12)	0.0090 (14)	0.0009 (13)

### Geometric parameters (Å, °)

**Table:**

Br1—C3	1.897 (3)	C5—C6	1.391 (4)
O1—C10	1.245 (3)	C5—H5	0.9500
N1—C10	1.332 (3)	C6—C7	1.537 (4)
N1—H1A	0.886 (9)	C7—C9	1.536 (4)
N1—H1B	0.882 (9)	C7—C8	1.540 (4)
C1—C2	1.373 (4)	C7—C10	1.547 (4)
C1—C6	1.396 (4)	C8—H8A	0.9800
C1—H1	0.9500	C8—H8B	0.9800
C2—C3	1.387 (4)	C8—H8C	0.9800
C2—H2	0.9500	C9—H9A	0.9800
C3—C4	1.387 (4)	C9—H9B	0.9800
C4—C5	1.383 (4)	C9—H9C	0.9800
C4—H4	0.9500		
			
C10—N1—H1A	125.1 (16)	C9—C7—C6	109.3 (2)
C10—N1—H1B	117.5 (15)	C9—C7—C8	108.9 (2)
H1A—N1—H1B	117.2 (16)	C6—C7—C8	112.7 (2)
C2—C1—C6	121.5 (3)	C9—C7—C10	109.2 (2)
C2—C1—H1	119.2	C6—C7—C10	107.4 (2)
C6—C1—H1	119.2	C8—C7—C10	109.3 (2)
C1—C2—C3	119.7 (3)	C7—C8—H8A	109.5
C1—C2—H2	120.1	C7—C8—H8B	109.5
C3—C2—H2	120.1	H8A—C8—H8B	109.5
C4—C3—C2	120.0 (3)	C7—C8—H8C	109.5
C4—C3—Br1	120.3 (2)	H8A—C8—H8C	109.5
C2—C3—Br1	119.6 (2)	H8B—C8—H8C	109.5
C5—C4—C3	119.5 (3)	C7—C9—H9A	109.5
C5—C4—H4	120.3	C7—C9—H9B	109.5
C3—C4—H4	120.3	H9A—C9—H9B	109.5
C4—C5—C6	121.5 (3)	C7—C9—H9C	109.5
C4—C5—H5	119.3	H9A—C9—H9C	109.5
C6—C5—H5	119.3	H9B—C9—H9C	109.5
C5—C6—C1	117.7 (3)	O1—C10—N1	121.1 (3)
C5—C6—C7	122.4 (3)	O1—C10—C7	121.6 (2)
C1—C6—C7	119.9 (3)	N1—C10—C7	117.3 (2)
			
C6—C1—C2—C3	0.0 (5)	C1—C6—C7—C9	−78.0 (3)
C1—C2—C3—C4	−3.2 (5)	C5—C6—C7—C8	−19.2 (4)
C1—C2—C3—Br1	173.2 (2)	C1—C6—C7—C8	160.7 (2)
C2—C3—C4—C5	3.1 (5)	C5—C6—C7—C10	−139.6 (3)
Br1—C3—C4—C5	−173.2 (2)	C1—C6—C7—C10	40.3 (3)
C3—C4—C5—C6	0.1 (5)	C9—C7—C10—O1	14.6 (4)
C4—C5—C6—C1	−3.1 (4)	C6—C7—C10—O1	−103.8 (3)
C4—C5—C6—C7	176.8 (3)	C8—C7—C10—O1	133.6 (3)
C2—C1—C6—C5	3.0 (4)	C9—C7—C10—N1	−165.7 (2)
C2—C1—C6—C7	−176.8 (3)	C6—C7—C10—N1	75.9 (3)
C5—C6—C7—C9	102.1 (3)	C8—C7—C10—N1	−46.6 (3)

### Hydrogen-bond geometry (Å, °)

**Table:**

*D*—H···*A*	*D*—H	H···*A*	*D*···*A*	*D*—H···*A*
N1—H1A···O1^i^	0.89 (1)	2.12 (1)	2.990 (3)	167 (3)
N1—H1B···O1^ii^	0.88 (1)	2.12 (1)	3.002 (3)	173 (3)

Symmetry codes: (i) *x*, −*y*+1/2, *z*−1/2; (ii) −*x*, −*y*+1, −*z*+2.
